# A haplotype inference algorithm for trios based on deterministic sampling

**DOI:** 10.1186/1471-2156-11-78

**Published:** 2010-08-23

**Authors:** Alexandros Iliadis, John Watkinson, Dimitris Anastassiou, Xiaodong Wang

**Affiliations:** 1 Center for Computational Biology and Bioinformatics and Department of Electrical Engineering, Columbia University, New York, NY, USA

## Abstract

**Background:**

In genome-wide association studies, thousands of individuals are genotyped in hundreds of thousands of single nucleotide polymorphisms (SNPs). Statistical power can be increased when haplotypes, rather than three-valued genotypes, are used in analysis, so the problem of haplotype phase inference (phasing) is particularly relevant. Several phasing algorithms have been developed for data from unrelated individuals, based on different models, some of which have been extended to father-mother-child "trio" data.

**Results:**

We introduce a technique for phasing trio datasets using a tree-based deterministic sampling scheme. We have compared our method with publicly available algorithms PHASE v2.1, BEAGLE v3.0.2 and 2SNP v1.7 on datasets of varying number of markers and trios. We have found that the computational complexity of PHASE makes it prohibitive for routine use; on the other hand 2SNP, though the fastest method for small datasets, was significantly inaccurate. We have shown that our method outperforms BEAGLE in terms of speed and accuracy for small to intermediate dataset sizes in terms of number of trios for all marker sizes examined. Our method is implemented in the "Tree-Based Deterministic Sampling" (TDS) package, available for download at http://www.ee.columbia.edu/~anastas/tds

**Conclusions:**

Using a Tree-Based Deterministic sampling technique, we present an intuitive and conceptually simple phasing algorithm for trio data. The trade off between speed and accuracy achieved by our algorithm makes it a strong candidate for routine use on trio datasets.

## Background

Large genetic association studies involving thousands of individuals are becoming increasingly available, providing opportunities for biological and medical discoveries using sophisticated computational and statistical analysis [1]. Typically, individuals are genotyped using high throughput platforms so that each of hundreds of thousands of single nucleotide polymorphisms (SNPs) is assigned one of three values: homozygous major, homozygous minor, and heterozygous.

Rather than examining SNPs independent of each other, simultaneously considering the values of multiple SNPs within haplotypes (combinations of alleles at multiple loci in individual chromosomes) can improve the power of detecting associations with disease and is helpful in several applications, such as evolutionary genetics [2-6]. Since there are numerous haplotype arrangements for heterozygous SNPs that are consistent with the available three-level genotyped values, the problem of inferring haplotype phase ("phasing") becomes particularly relevant. Such inference is based on modelling the mechanisms and the biological processes generating sequence variation. Associated computational and statistical techniques can be used on population samples, based on parsimony, the Hardy-Weinberg principle that allele and genotype frequencies in a population remain constant in equilibrium, Markov chain Monte Carlo, hidden Markov models, Expectation Minimization (EM) algorithm, etc. Most algorithms are designed to be generally used for unrelated individuals. Use of pedigree information [7], if available, is useful. In particular, "trio" data consisting of genotypes given in father-mother-child triplets are obtained in genome-wide association studies and some phasing algorithms are adapted to be used in this type of data.

An algorithm (PHASE [8]) used in the HapMap project [9] uses a Bayesian approach attempting to capture the tendency that haplotypes cluster together over regions of the chromosome and that this clustering can change as we move along the chromosome because of recombination. It uses a flexible model for the decay of linkage disequilibrium (LD, the non-random association of alleles) with distance. Although PHASE is considered the most accurate method, its computational complexity makes it prohibitively slow even for intermediate-sized datasets. Thus, it may not be the method of choice for routine use in large genome-wide association studies. On the other extreme of the trade-off between complexity and accuracy, a computationally simple method (2SNP [10]) uses maximum spanning trees to successively phase whole genotypes starting from SNP pairs. Other well known approaches include HAP [11], using imperfect phylogeny, HAP2 using a Markov Chain Monte Carlo (MCMC) scheme [12] and PL-EM [13], which uses an Expectation Maximization (EM) algorithm. A Gibbs sampling method, Haplotyper, is proposed in [14], which introduces the partition-ligation (PL) method to support haplotype inference on long genotype vectors, a procedure adopted by some of the aforementioned methods so that they can be extended to large datasets. An obvious problem of the Gibbs sampler and of most of the previous frameworks is that when new data is introduced into the original dataset, the previous data also has to be reused in the estimation of the new data. Another drawback of using Gibbs sampler and EM algorithm in the haplotype inference problem is the lack of robustness of these two algorithms when the parameter space exhibits multimodality such as the one we encounter in the haplotype inference problem [15]. The performance of these methods has been evaluated in simulated datasets of both trio as well as unrelated individuals in a comparative review [16], providing some "gold standard" datasets for future algorithms to be compared upon. A more recent approach (BEAGLE [17,18]) uses localized haplotype clustering and fits the data using an EM-style update.

It is important for phasing methods that they scale well with the number of SNPs as well as the number of individuals. It is also important in terms of computational time that when new data is inserted in phased datasets, we do not have to reuse the previous data in the estimation of the new data. A deterministic sequential Monte Carlo (DSMC) - based phasing algorithm [19] has recently been proposed for unrelated individuals, allowing for large datasets. It jointly infers haplotype pair solutions and estimates haplotype frequencies based on Hardy-Weinberg equilibrium. It also uses a partition ligation method to allow processing of large SNP sets.

In this paper, we propose a related new TDS algorithm for haplotype phasing of trio data, in which trios are processed sequentially. All possible solutions for each haplotype are examined. Our algorithm uses the idea that within haplotype blocks there is limited haplotype diversity and thus attempts to phase each new trio using haplotypes that have already been encountered in the previously seen trios. The TDS framework allows us to effectively perform this search in the space of all possible solution combinations. The procedure can be seen as an efficient tree search procedure where in each step only "the most probable" solution streams are kept. Each of them contains one and only one solution for each trio already encountered. We show that our algorithm demonstrates an excellent tradeoff of speed and accuracy, making it ideal for routine use.

## Results

The structure of this section is as follows: First we describe the datasets and figures of merit used to evaluate the method. Then we present the results from comparing our method to PHASE v2.1, BEAGLE v3.0.2 and 2SNP v1.7.

### Datasets

We used a set of simulated datasets produced with the "COSI" software as provided in [16]. The haplotypes were simulated using a coalescent model that incorporates variation in recombination rates and demographic events and the parameters of the model were chosen to match aspects of data from a sample of Caucasian Americans [20,16]. Three classes of dataset were provided, with each consisting of 20 sets of 30 trios spanning 1 Mb of sequence with a density of 1 SNP per 5 kb [16].

We also used the "COSI" software to create our own realistic simulated data sets to assess the performance of our method on large datasets. We created 20 datasets, each of them consisting of 4000 haplotypes with 20 Mb of marker data using the "best-fit" parameters obtained from fitting a coalescent model to the real data. Samples were taken from a European population and each simulated data set has a recombination rate sampled from a distribution matching the deCODE map, with recombination clustered into hotspots. For each simulated data set, we initially selected only those markers with minor allele frequency greater than 0.05. Markers were then randomly selected to obtain a density of about 1 SNP per 3 kb. In each dataset two sample sizes were created: 100 and 1000 trios. In each trio, each parent was randomly assigned a haplotype from the population so that no two individuals had the same haplotype and one of the haplotypes of each parent was selected to be transmitted to the child.

### Definitions of criteria

Transmission Error Rate: The proportion of non-missing parental genotypes with ambiguous phase that were incorrectly phased [18].

Incorrect Trios (IT): The number of trios for which phasing was not completely correct.

Computational Time: The average time to complete phasing. Our algorithm was implemented in Java for portability, memory efficiency and speed. For each method we recorded the average computational time in each dataset on a 3.66 GHz Xeon Intel PC with 8 GB of RAM.

Memory: The memory required by the software to complete haplotype inference.

### Transmission Error Rate and Incorrect Trios

The performance of the methods on the simulated data sets is shown in Tables 1 and 2. We decreased the <nsamples> parameter in BEAGLE from the default value, *R = 4*, to decrease computational time. Our purpose was to make the results of BEAGLE and TDS as comparable as possible by allowing both methods to run for approximately the same time. PHASE shows superior performance to all other methods in all datasets for both figures of merit. 2SNP was consistently outperformed by all other methods, consistent with the result mentioned in [17]. For most of the datasets, a lower transmission error rate usually implied fewer incorrectly-phased individuals. TDS shows superior performance to BEAGLE and 2SNP for all datasets, losing only to PHASE.

**Table 1:** Average Transmission Error Rate For Phasing Trios

	Average Transmission Error Rate (%)		
	ST1	ST2	ST3
PHASE	0.0013	0.0013	0.0145
BEAGLE			
R = 1	0.0235	0.0318	0.0426
R = 4	0.0150	0.0148	0.0344
TDS	0.0039	0.0065	0.0320
2SNP	0.4377	0.4868	0.4861

**Table 2:** Average number of Incorrect Trios per dataset

	Incorrect Trios		
	ST1	ST2	ST3
PHASE	0.3	0.4	2.45
BEAGLE			
R = 1	3.75	5.8	6.4
R = 4	1.95	2.9	5.45
TDS	0.95	1.6	5.4
2SNP	25.9	28.6	28

We set 1% of the genotypes to missing values and we re-evaluated the performance of the algorithms in these datasets and the results are shown in Tables 3 and 4. We again see that TDS shows superior performance compared to BEAGLE with <nsamples> parameter equal to 1 on all datasets. When we set in BEAGLE <nsamples > = 4, BEAGLE shows superior performance on the ST3 dataset and marginally on ST1 dataset.

**Table 3:** Average Transmission Error Rate For Phasing Trios with 1% Missing Rate

	Average Transmission Error Rate (%)		
	ST1	ST2	ST3
PHASE	0.0031	0.0023	0.0161
BEAGLE			
R = 1	0.0213	0.0248	0.0354
R = 4	0.0093	0.0133	0.0278
TDS	0.0094	0.0116	0.0348
2SNP	0.3038	0.3486	0.3169

**Table 4:** Average number of Incorrect Trios per dataset with 1% Missing Rate

	Incorrect Trios		
	ST1	ST2	ST3
PHASE	0.6	0.475	2.653
BEAGLE			
R = 1	3.6054	5.25	6.4661
R = 4	1.7464	3.1321	4.8893
TDS	1.7521	2.7018	5.7768
2SNP	26.05	28.55	28.2

We demonstrated the accuracy of our method with increasing dataset size by varying the number of trios and markers and evaluated the performance by means of the Transmission Error Rate as shown in Table 5. We used marker sizes of 200, 400, 1000 and 6000 markers for 100 and 1000 trios. Due to the excessive computational time of PHASE, we excluded it from these comparisons. Furthermore, we avoided using the number of Incorrect Trios as means of comparison, because as the genotype vectors grow longer, eventually all methods will find it hard to correctly infer the entire haplotype and the number of Incorrect Trios will be the total number of trios. For datasets of the size of 1000 trios we noted that, in order to be able to take advantage of the information offered as a whole, we had to allow a very large number of streams in our algorithm (Methods section) that would result in excessive computational time. However, we found that we could have minor losses by partitioning the dataset in slices of 100 trios where we had established significant gain compared to BEAGLE. From Table 5 we see that TDS shows superior performance for datasets of up to 100 trios for all marker sizes. For datasets of the size of 1000 trios, BEAGLE showed superior performance to all methods.

**Table 5:** Average Transmission error rate for 100 and 1000 Trios as a function of the number of markers

		Markers			
		200	400	1000	6000
TDS	100	0.00063	0.00075	0.0015	0.0023
	1000	0.00042	0.0008	0.0015	0.0023

Beagle	100	0.0013	0.0013	0.0021	0.0024
	1000	0.00011	0.00033	0.0005	0.0007

2SNP	100	0.1094	0.2855	0.3916	0.4315
	1000	0.1733	0.2524	0.3836	0.4117

### Timing Results

The computational times for datasets ST1, ST2 and ST3 are displayed in Table 6. In Table 7 we present the average running time on the same datasets, but with randomly inserting 1% missing SNPs in each one of them. Based on these times 2SNP is the fastest algorithm followed by TDS. Both algorithms were faster than the fastest BEAGLE runs done with <nsamples> parameter equal to 1. PHASE was the slowest algorithm with computational times 3 orders of magnitude more than the remaining three algorithms.

**Table 6:** Timing Results

	Time(s)		
	ST1	ST2	ST3
PHASE	8452	4932	5464
BEAGLE			
R = 1	2.59	2.73	2.95
R = 4	2.80	3.18	3.27
TDS	1.99	2.48	2.61
2SNP	0.63	0.6	0.59

**Table 7:** Timing Results with 1% Missing Rate

	Time(s)		
	ST1	ST2	ST3
PHASE	8613	5220	5831
BEAGLE			
R = 1	2.6744	2.9873	3.2409
R = 4	2.9233	3.2858	3.4429
TDS	2.0643	2.5815	2.7484
2SNP	0.67	0.63	0.6

In Table 8 we demonstrate that for large datasets TDS scaled almost linearly with the number of markers and, as described in the previous subsection, with the number of trios. For datasets of up to 100 trios, our method is faster than BEAGLE; however for datasets of 1000 trios, BEAGLE is the fastest of all methods for marker sizes up to 400 markers.

**Table 8:** Average Timing Results in seconds for 100 and 1000 Trios as a function of the number of markers

		Markers			
		200	400	1000	6000
TDS	100	2.8	5	14.4	113.6
	1000	31.8	63.3	156.2	1257.4

Beagle	100	3.7	5.6	15.2	118.4
	1000	12.7	31.6	291.8	1952.4

2SNP	100	3	8.9	28.7	180.7
	1000	33.4	116.2	399.8	3008.2

### Memory Requirements

All methods could complete the experiments within the preallocated 1.5 Gb of RAM.

## Discussion

An important feature of our algorithm is the partition of the whole genotype sequence in smaller blocks that exhibit limited haplotype diversity. We currently identify these haplotype blocks based on the genotype sequences (see Haplotype Block Partitioning section). However, we can have significant gain in the accuracy of our algorithm if we improve the accuracy in the estimation of the boundaries of the haplotype blocks. To achieve that, either the haplotype blocks should be already known from outside sources, or a set of phased haplotypes from the region at interest should be already available. In real applications, it is very often the case that studies are performed in populations that are already studied in the HapMap project. This means that for these populations we have accurately phased samples, which can be used as a basis for accurate definition of the haplotype blocks. Our methodology offers a unique framework that can easily incorporate prior knowledge in the form of haplotypes or trio genotypes from the same population as that from which the target samples were drawn. In the case of haplotypes (such as those available from the HapMap), they are introduced in the form of a prior for the counts in the TDS algorithm. In the case of unphased trio genotypes, the trios can be phased along with the target samples, with the result discarded at the end. The presence of the extra information will improve the phasing accuracy on the target samples.

A related problem to haplotype inference is imputation of missing SNPs. Several algorithms have been specifically developed to address this problem [21]. Some of the aforementioned algorithms have been extended and configured to complex imputation scenarios involving the use of prior information (in the form of known phased samples or extra genotype samples) for performing imputation in markers not typed in the original samples.

In datasets with missing SNPs such as the ones used in Tables 3, 4 and 7 the imputation of the missing values is done internally in most phasing algorithms so that phasing can be performed. Many haplotype inference algorithms are used to that extent and on a regular basis on this simple and common imputation scenario. Therefore, to provide a complete description of our algorithm from the user perspective, and at the same time show the potential applicability of our framework to the missing SNP imputation problem, we have evaluated the allelic-imputation error rate[18], defined as the proportion of mistakenly inferred alleles among all missing alleles. We have used two kinds of datasets. First, we have evaluated the allelic-imputation error rate on the simulated datasets used in Tables 3, 4 and 7 and the results are shown in Table 9. We have also created 20 real datasets from the CEU HapMap [22] population(HapMap 3 release 2). Each dataset consists of 44 trios. The datasets were created by randomly choosing 20 1 MB regions across the genome. In each region we initially selected only those markers with minor allele frequency greater than 0.05 and then randomly selected markers to obtain a density of approximately a marker per 5 kb. In each dataset we set 1% of the genotypes to missing values and evaluated the performance in terms of the allelic imputation error rate and running time. The results are shown in Table 10. TDS is the second fastest algorithm after 2SNP for all datasets with PHASE showing superior performance to all algorithms in terms of the allelic imputation error rate and TDS showing performance close to BEAGLE.

**Table 9:** Average Allelic Imputation Error Rate For Simulated datasets

	Average Allelic Imputation Error Rate (%)		
	ST1	ST2	ST3
PHASE	0.0063	0.0145	0.0133
BEAGLE			
R = 1	0.0124	0.0255	0.0249
R = 4	0.0101	0.0224	0.0223
TDS	0.0124	0.0271	0.0266
2SNP	0.0741	0.0855	0.0983

**Table 10:** Average Allelic Imputation Error Rate and Timing Results for HapMap datasets

	Allelic Imputation Error Rate	Time(s)
PHASE	0.0051	5360
BEAGLE		
R = 1	0.0129	3.156
R = 4	0.0112	3.339
TDS	0.0134	2.53
2SNP	0.0831	0.685

## Conclusions

We have introduced a new algorithm for inferring haplotype phase in nuclear families using a Tree-Based Deterministic sampling scheme. PHASE, which is the most accurate algorithm for haplotype inference in trio families, is prohibitively slow for routine use, and 2SNP, which is the fastest algorithm for datasets up to 100 trios, is inaccurate. We have demonstrated that TDS is faster and more accurate than BEAGLE in almost all scenarios considered in small and intermediate dataset sizes in terms of trios and for all marker sizes. From a user's perspective, our implementation is friendlier since it is parameter - free, as all parameters are optimized inside the algorithm. This makes it ideal for routine tasks even for non specialized users. Furthermore, our TDS implementation provides a comprehensive, solid and straightforward framework to build upon for more complex phasing and imputation scenarios.

## Methods

### Brief Description

We first give an intuitive description of our algorithm highlighting its major concepts without going into detailed mathematical formalization. Suppose that we denote the major allele in a particular SNP locus in a haplotype as "0" and the minor allele as "1". Similarly in a genotype we denote by 0 that the individual is homozygous to the major allele at that SNP and with "1" that the individual is homozygous to the minor allele. We denote by "2" the heterozygous case. For example, the haplotype pair "10110" and "00100" would produce the genotype "20120".

**Figure 1:**
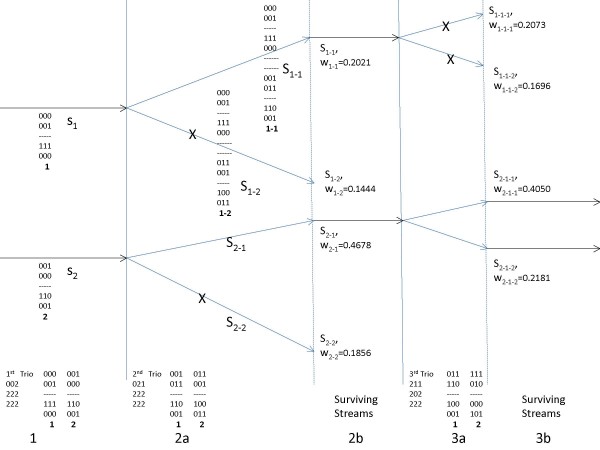
**Example of TDS**. We process three trios sequentially. In each trio the first two genotypes are the genotypes of the parents and the third genotype is the genotype of the child. The possible solutions of each trio are given exactly next to it and numbered 1, 2. In each of the possible solutions for each trio the first two genotypes are the transmitted and the untransmitted haplotype from the first parent and similarly the remaining two for the second parent. At each step we are willing to keep only K = 2 streams which would be called "surviving streams". 1) The first trio has two possible solutions. 2) a) The second trio has two possible solutions. We have four possible combinations of a solution from the first trio to a solution from the second. The indices below the solutions show from which solutions from each trio this stream was created. For example stream s_1-2 _as illustrated, was created from the first solution in the first trio and from the second in the second. In each stream we associate a weight as described in method section. b) We keep only the K = 2 streams with the highest weights (surviving streams) so at this point we consider them as the most probable and keep them. 3) The third trio has 2 possible solutions. a) Each one of them is appended in the end of each of the two solutions that we have kept. The definition of the streams is similar as before with stream s_2-1-1 _coming from appending solution 1 of the third trio to stream s_2-1_. b) Again we keep only two of the streams the ones with the highest weights s_2-1-1 _and s_2-1-2._

In nuclear families, each parent transmits a chromosome to a child. In most cases we can detect which parent transmitted which SNP to the offspring based on the genotypes of the parents and the offspring. The only case where we cannot infer that information is when both parents and the offspring are all heterozygous to that SNP (i.e., at that SNP all three genotypes are "2"). In that case, either parent can have transmitted the major or the minor allele, so we have two possibilities for the origin of each allele. This means that if a genotype of a trio has *L *ambiguous SNPs, then this trio would have 2*^L ^*possible solutions (see solutions for the trios in Figure 1).

Our algorithm processes nuclear families sequentially (Figure 1). In each family, multiple solutions are produced when we encounter a triple heterozygote SNP as explained earlier. Our algorithm examines all these different possible solutions.

Suppose we had *n *trios and each one of them had {*K_l_*, ..., *K_n_*} possible solutions. If we evaluate simultaneously all solutions for all haplotypes, which would obviously be the optimal way, we would end up with a total of $\prod_{i=1}^{n}K_{i}$ possible solutions each one of them having one and only one solution for each trio. To be consistent with the remaining sections we will call "solution" only the final solution and we will call these potential solutions as solution streams. Clearly this number of solution streams would be infeasible for all non trivial applications. Instead, in our algorithm we process trios sequentially and after processing each trio we keep only a pre-specified *K *number of solution streams that would be the most probable ones (Figure 1-2b and 1-3b keeping only *K *= 2 streams in the end of these steps). Each one of these streams would have one and only one solution for each trio we have encountered (Figure 1).

To further explain this procedure, suppose that after processing a trio we have *K *streams. When the next trio is processed, which has, say, *K^ext ^*possible solutions, we append each of these solutions to each of the previous *K *streams resulting in a total of *K *× *K^ext ^*streams (Figure 1-2a and 1-3a). From these streams we keep only the *K *most probable ones (Figures 1-2b and 1-3b). So we always end up with *K *streams after processing each trio.

The idea for weighting the different streams is based on the concept that within a haplotype block we expect to have limited diversity and find only a subset of all the possible haplotypes. This means that most haplotypes should be encountered more than once. In terms of our procedure we would like to phase each new trio based on haplotypes that we have already encountered in that stream. Since the weight we assign to each node should capture this feature, it is a function of the weight that this node had prior to attaching one of the possible solutions of the current trio and of a factor that represents how the currently appended solution includes haplotypes that have already been seen (see Eq. (4) in Methods section).

### Definitions and Model Selection

Let us assume that we have *N *subjects genotyped in *L *SNPs. Suppose that *g*_*t *_are the genotypes of the *t*^th ^trio, i.e., *g_t _*= {*g_t,f_, g_t,m_, g_t,c_*} where *g_t,f_, g_t,m_, g_t,c _*are the genotypes of the father the mother and the child of trio *t *respectively. Suppose also that *G_t _*= {*g*_1_, ..., *g_t_*} is a set of genotypes of trios up to and including trio *t*. In each trio we consider the haplotypes of the parents denoted as *h_t _*= {*h*_*t*,1_, *h*_*t*,2_, *h*_*t*,3_, *h*_*t*,4_}, where {*h*_*t*,1_, *h*_*t*,2_} are the two haplotypes of the first parent and {*h*_*t*,3_, *h*_*t*,4_} are the two haplotypes of the second parent and similarly define *H_t _*= {*h*_1_, ..., *h_t_*} Let us also define as *θ *= {*θ*_1_, ..., *θ_M _*} a set of population haplotype frequencies for all the *M *haplotypes that appear in the population and *Z *= {*z*_1_, ..., *z_y_*} as the set of haplotypes compatible with at least a genotype of any trio.

Let us consider the following dynamic model

• Initial state model *p_θ _*(*h*_0_)

• State transition model *p_θ _*(*h_t_*|*h*_*t*-1_) for *t *≥ 1

• Measurement model *p_θ _*(*g_t_*|*h_t_*) for *t *≥ 1

where *p_θ_*(•) are probability density functions depending on some unknown parameters *θ*.

In the next subsection, for the convenience of the reader, we present the form that the system update equations would have should the system parameters were known. Then we move forward and make the connection to the real scenario were the system parameters are not known.

### TDS ESTIMATOR with known system parameters θ

We assume that by the time we have processed genotype *g_t-1 _*we have a set of solution streams and their associated weights $\left\{(H_{t-1}^{\left(k\right)}|w_{t-1}^{\left(k\right)}),k=1,...,K\right\}$ properly weighted with respect to the posterior distribution *p_θ _*(*H*_*t*-1_|*G*_*t*-1_). When we process the individual *t *we would like to make an online inference of the haplotypes *H_t _*based on the genotypes *G_t_*. From Bayes' theorem we have

(1) $$\begin{array}{l} p_{\theta }(H_{t}|G_{t})\\ \propto p_{\theta }(g_{t}|H_{t},G_{t-1})p_{\theta }(H_{t}|G_{t-1})\\ \propto p_{\theta }(g_{t}|H_{t},G_{t-1})p_{\theta }(h_{t}|H_{t-1},G_{t-1})\\ \times p_{\theta }(H_{t-1}|G_{t-1}) \end{array}$$

Given the set of solution streams and the associated weights we approximate the distribution *p_θ _*(*H*_*t*-1_|*G*_*t*-1_) as follows:

$${\overset{\wedge }{p}}_{\theta }(H_{t-1}|G_{t-1})=\frac{1}{W_{t-1}}\sum_{k=1}^{K}w_{t-1}^{\left(k\right)}I(H_{t-1}-H_{t-1}^{\left(k\right)})$$

where $W_{t-1}=\sum_{k=1}^{K}w_{t-1}^{\left(k\right)},$

and *I*(•) is the indicator function such that I(*x *- *y*) = 1 for *x *= *y *and I(*x *- *y*) = 0 otherwise.

From the previous relationships, if we knew the system parameters *θ*, and assuming that there are *K^ext ^*possible haplotypes compatible with the genotype of the *t*^th ^trio, we would be able to approximate the distribution of *p_θ _*(*H_t_*|*G_t_*) as

$$\begin{array}{l} {\overset{\wedge }{p}}_{\theta }(H_{t}|G_{t})=\frac{1}{W_{t}^{ext}}\\ \times \sum_{k=1}^{K}\sum_{i=1}^{Kext}w_{t}^{\left(k,i\right)}I(H_{t}-[H_{t-1}^{\left(k\right)},h_{t}^{\left(i\right)}]) \end{array}$$

where [$H_{t-1}^{\left(k\right)},h_{t}^{\left(i\right)}$] represents the vector obtained by appending the element $h_{t}^{\left(i\right)}$ to the vector $H_{t-1}^{\left(k\right)}$

and $W_{t}^{ext}=\sum_{i,k}w_{t}^{\left(k,i\right)}$ with

$$w_{t}^{\left(k,i\right)}\propto w_{t-1}^{\left(k\right)}p_{\theta }(g_{t}|h_{t}=i)p_{\theta }(h_{t}=i|H_{t-1}^{\left(k\right)}).$$

### TDS Estimator with unknown system parameters θ

However, the system parameters are not known. Suppose now that their posterior distribution given *H*_*t *_and *G*_*t *_only depends on a set of sufficient statistics *T_t _*= *T_t _*(*H_t_*|*G_t_*) = *T_t _*(*T*_*t*-1_, *h_t_*, *g_t_*).

Similarly to (1) we have:

(2) $$\begin{array}{l} p_{\theta }(H_{t}|G_{t},Z)\\ \propto p_{\theta }(g_{t}|H_{t},G_{t-1})\\ \times p_{\theta }(h_{t}|H_{t-1},G_{t-1})p_{\theta }(H_{t-1}|G_{t-1},Z)\\ \propto p_{\theta }(H_{t-1}|G_{t-1},Z)\\ \times \int p(g_{t}|h_{t},\theta )p(\theta |h_{t},H_{t-1},G_{t-1},Z)d\theta \\ \times \int p(h_{t}|H_{t-1},\theta ,Z)p(\theta |T_{t-1},Z)d\theta \\ \propto p_{\theta }(H_{t-1}|G_{t-1},Z)\\ \times \int p(h_{t}|H_{t-1},\theta ,Z)p(\theta |T_{t-1},Z)d\theta \end{array}$$

Conditional on the haplotype of the *t*^th ^trio the genotype of that trio is unique and is independent of all the previous observations *G_t-1 _*and haplotypes *H_t-1 _*that we have seen. So the term *p_θ _*(*g_t_*|*H_t_,G*_*t*-1_) and consequently the integral ∫ *p*(*g_t_*|*h_t_,θ*)*p*(*θ*|*h_t_*, *H*_*t*-1_, *G*_*t*-1_, *Z*)*dθ *are zero if the genotype is not compatible with haplotype *h_t _*and 1 otherwise.

The recursion now lies only in computing the integral in (2).

In order to calculate the integral in the previous equation we will define the prior distribution for the parameters *θ *and we will show how to update their posterior distribution.

### Prior and Posterior Distribution for θ

Assuming random mating in the population it is clear that the number of each unique haplotype in *H *is drawn from a multinomial distribution based on the haplotype frequency *θ *[23]. Using the same reasoning as [19] it leads us to the use of the Dirichlet distribution as the prior distribution for *θ *so that

$$\theta \sim D(\rho_{1},\ldots ,\rho_{M})$$

With mean

$$E\{\theta_{i}\}=\frac{\rho_{i}}{\sum_{j=1}^{M}\rho_{\text{j}}}$$

Next we will show that the posterior distribution for *θ *is also Dirichlet and we will calculate its parameters. As we have also noted before, the emission probabilities *p_θ _*(*g_t_*|*h_t_*) do not depend on the parameters *θ*, and they are zero if the genotype vector of the trio is not compatible with the haplotype and 1 otherwise.

$$\begin{array}{l} p(\theta |G_{t},H_{t},Z)\\ \propto p(g_{t}|h_{t}=(h_{t,1},h_{t,2},h_{t,3},h_{t,4}),\theta ,G_{t-1},H_{t-1})\\ \times p(h_{t}=(h_{t,1},h_{t,2},h_{t,3},h_{t,4})|\theta ,G_{t-1},H_{t-1},Z)\\ \times p(\theta |G_{t-1},H_{t-1})\\ \propto p(h_{t}=(h_{t,1},h_{t,2},h_{t,3},h_{t,4})|\theta ,Z)\\ \times p(\theta |G_{t-1},H_{t-1},Z)\\ \propto \theta_{h_{t,1}}\theta_{h_{t,2}}\theta_{h_{t,3}}\theta_{h_{t,4}}\prod_{m=1}^{M}\theta_{m}^{\rho_{m}(t-1)-1}\\ \propto \prod_{m=1}^{M}\theta_{m}^{\rho_{m}(t-1)-1+\sum_{i=1}^{4}I(z_{m}-h_{t,i})}\\ \propto D(\rho_{1}(t-1)\\ +\sum_{i=1}^{4}I(z_{1}-h_{t,i}),...,\rho_{M}(t-1)+\sum_{i=1}^{4}I(z_{M}-h_{t,i}) \end{array}$$

where we denote *ρ_m_*(*t*) *m = 1,...,M *as the parameters of the distribution of *θ *after the *t*^th ^trio and *I(z_m _*- *h_t,i_*) with *i = 1,...,4 *is the indicator function which equals 1 when *z_m _*- *h_t,i _*is a vector of zeros, and 0 otherwise.

### TDS-Estimator

We have that *p*(*θ*|*T*_*t*-1_) = *D*(*θ*; *p*_1_(*t*-1), ..., *p_M_*(*t*-1)) and also that

$$\begin{array}{c} p(h_{t}=(h_{t,1},h_{t,2},h_{t,3},h_{t,4})|H_{t-1},\theta ,Z)\\ =\theta_{h_{t,1}}\theta_{h_{t,2}}\theta_{h_{t,3}}\theta_{h_{t,4}} \end{array}$$

and therefore we can calculate the integral in (2) as follows:

$$\begin{array}{l} \int p(h_{t}=(h_{t,1},h_{t,2},h_{t,3},h_{t,4})|\theta ,Z)\\ \times p(\theta |T_{t-1},Z)d\theta \\ =E_{\theta |T_{t-1}}\{\theta_{h_{t,1}}\theta_{h_{t,2}}\theta_{h_{t,3}}\theta_{h_{t,4}}\}\\ =\frac{\rho_{h_{t,1}}(t-1)\rho_{h_{t,2}}(t-1)\rho_{h_{t,3}}(t-1)\rho_{h_{t,4}}(t-1)}{{\left(\sum_{m=1}^{M}\rho_{m}(t-1)\right)}^{4}} \end{array}$$

where $\rho_{h_{t,i}}(t-1)=\{\rho_{z_{m}}(t-1):h_{t,i}=z_{m}\}$

Having calculated the integral, we can go back to the recursion and assuming that we have approximated *p*(*H*_*t*-1_|*G*_*t*-1_), we can approximate *p*(*H_t_*|*G_t_*) as

(3) $$\begin{array}{l} {\overset{\wedge }{p}}^{ext}(H_{t}|G_{t})=\frac{1}{W_{t}^{ext}}\\ \times \sum_{k=1}^{K}\sum_{i=1}^{Kext}w_{t}^{\left(k,i\right)}I(H_{t}-[H_{t-1}^{\left(k\right)},h_{i,1},h_{i,2},h_{i,3},h_{i,4}]) \end{array}$$

The weight update formula is given by

(4) $$w_{t}^{\left(k,j\right)}\propto w_{t-1}^{\left(k\right)}\frac{\rho_{h_{j,1}}^{\left(k\right)}(t-1)\rho_{h_{j,2}}^{\left(k\right)}(t-1)\rho_{h_{j,3}}^{\left(k\right)}(t-1)\rho_{h_{j,4}}^{\left(k\right)}(t-1)}{{\left(\sum_{m=1}^{M}\rho_{m}^{\left(k\right)}(t-1)\right)}^{4}}$$

### Haplotype Block Partitioning

Again, we use the idea that haplotypes exhibit block structures so that within each block the haplotype blocks exhibit limited diversity compared to the whole haplotype vectors. To define these blocks we use a Dynamic Programming (DP) algorithm similar to the one used in [19] so that we partition *G *into subsets of genotype segments. Our criterion for the DP algorithm partition would be that the sum of the entropies of the genotypes of the individual blocks would be minimum.

Let us define *C *(*j*) as the minimum total block entropy up to the *j*^th ^SNP, where total block entropy is the sum of the entropies of all the blocks. If *G_i:j _*is the set of genotypes that contains genotype segments from SNP *i *to SNP *j*, the entropy *E *(*i,j*) of that segment can be computed from the number of occurrences of each unique genotype segment in *G_i:j _*.

More specifically if there are n distinct genotypes in *G_i:j_*, {g_1_, g_2_, ..., g_n_} each one of them with counts {a_1_, a_2_, ..., a_n_} then $E(i,j)=-\sum_{k=1}^{n}p_{k}\ln(p_{k})$, where ${\text{p}}_{\text{k}}=\frac{{\text{a}}_{\text{k}}}{\sum_{\text{l}=1}^{\text{n}}{\text{a}}_{\text{l}}}$. The DP algorithm then can be formulated as the following recursive structure:

$$C(j)=\underset{1\leq i\leq j}{\min}\{C(i-1)+E(i,j)\}$$

for *j *- *i *<*W*, where *W *is the maximum allowed haplotype block length.

When the DP algorithm was applied to the ST1,ST2 and ST3 datasets with the maximum allowed block size being 12, we obtained an average of 6 markers per block with the smallest block being a single marker and the largest equal to W. On average, we had 22 distinct haplotypes per block with their number ranging from 1 to 30.

Our algorithm is based on genotypes as opposed to haplotypes that were used in [19]. In the method proposed in [19], each genotype segment was first phased separately and the entropy of each block was calculated from the number of occurrences of each unique haplotype in that segment. The same DP algorithm was then applied to the segments and the minimum total block entropy partition was calculated. In order to avoid this time consuming procedure (it can result in computational times even bigger than PHASE) we create the blocks based on the genotypes that can be done instantly. Clearly the bigger the dataset the more accurate our genotype approximation results will be. However, even for small datasets this approach has been shown to improve our results compared to the standard equal block partitioning as shown in Tables 11 and 12.

**Table 11:** Average Transmission Error Rate for Equal Block Partitioning TDS (Equal TDS)

	Average Transmission Error Rate (%)		
	ST1	ST2	ST3
TDS	0.0039	0.0065	0.0320
Equal TDS	0.0113	0.0085	0.0360

**Table 12:** Average number of Incorrect Trios per dataset for Equal Block Partitioning TDS (Equal TDS)

	Incorrect Trio		
	ST1	ST2	ST3
TDS	0.95	1.6	5.4
Equal TDS	1.6	1.7	5.6

### Partition-Ligation

In the partition phase the dataset is divided into small segments of consecutive loci using the haplotype block partitioning method described above. Once the blocks are phased, they are ligated together using the following method (an extension of the original method described in [14]).

The result of phasing for each block is a set of haplotype solutions, paired with their associated weights. Two neighbouring blocks are ligated by creating merged solutions from all combinations of the block solutions, each associated with the product of the individual weights, called the *ligation weight*. The TDS algorithm is then repeated in the same manner as it was for the individual blocks. However, the weights of the solutions are scaled by the associated ligation weight for that solution. In this way, no information content is lost in the process of ligating.

Furthermore, the order in which the individual blocks are ligated is not predetermined. We first ligate the blocks that would produce in each step the minimum entropy ligation. This procedure allows us to ligate first the most homogenous blocks so that we have more certainty in the solutions that we produce while moving in the ligation procedure.

### Summary of the proposed algorithm

In the partition phase the dataset is divided into small segments of consecutive loci using the haplotype block partitioning.

**Routine 1**:

• Enumerate the set of all possible haplotype vectors, *Z*, based on the given dataset *G*.

• Initialization: Find all possible haplotype assignments for each trio and rearrange the trios in ascending order according to the number of distinct haplotype solutions each one of them has. Use the first *n *trios to enumerate all the possible streams, where *n *is the largest number such that the total number of streams enumerated from the *n *subjects does not exceed *K*, and compute their weights

• Update: For *i *= *n+1, n+2 ...*

∘ Find the *K^ext ^*possible haplotypes compatible with the genotype of the *i*^th ^trio.

∘ For *k = 1,2,..., K^ext^*

■ Enumerate all possible stream extensions $H_{i}^{\left(k,j\right)}=[H_{i-1}^{\left(k\right)},h_{j}]$ with *h_j _*= {*h*_*j*,1_, *h*_*j*,2_, *h*_*j*,3_, *h*_*j*,4_}

■ ∀*_j _*compute the weights $w_{i}^{\left(k,j\right)}$ according to (3)

∘ Select and preserve *K *distinct sample streams {$H_{i}^{\left(k\right)}$, *k = 1,...,K*} with the highest importance weights {$w_{i}^{\left(k\right)}$, *k = 1,...,K*} from the set {$H_{i}^{\left(k,j\right)},w_{i}^{\left(k,j\right)}$, *k = 1,...,K*, *j = 1,..., K^ext^*}

∘ ∀k, update the sufficient statistics $T_{i}^{\left(k\right)}=T_{i}(T_{i-1},h_{i}^{\left(k\right)},g_{i})$

TDS ALGORITHM

• Partition the genotype dataset *G *into *S *subsets using the procedure described in the "Haplotype-Block partitioning subsection".

• For *s = 1,...,S *apply Routine 1 so that all segments are phased and for each one keep all the solutions contained in the top *K *streams.

• Until all blocks are ligated

∘ Find the blocks that if ligated would produce the minimum entropy

∘ Ligate the blocks, following the procedure described in the Partition-Ligation section

## Authors' contributions

XW and DA conceived of the study. AI, JW, DA and XW participated in the design of the study. AI performed the computer experiments and wrote the first draft of the manuscript. All authors read and approved the final manuscript.
